# Smoking cessation in adolescents and effective strategies: a position paper

**DOI:** 10.1186/s13052-026-02281-y

**Published:** 2026-05-25

**Authors:** Antonio Corsello, Maria Elisa Di Cicco, Laura Reali, Michele Ghezzi, Laura Venditto, Valentina Agnese Ferraro, Mattia Spatuzzo, Rino Agostiniani, Luciana Indinnimeo, Stefania La Grutta

**Affiliations:** 1 Department of Clinical-Surgical, Diagnostic and Pediatric Sciences, University of Pavia, Pavia, Italy; 2 Children’s Hospital “G. Di Cristina”, ARNAS Civico-Di Cristina-Benfratelli, Palermo, Italy; 3 Section of Pediatrics, Department of Clinical and Experimental Medicine, University of Pisa, Pisa, Italy; 4 European Confederation of Primary Care Paediatricians, Lyon, France; 5 Pediatric Department, “Vittore Buzzi” Children’s Hospital, Milan, Italy; 6 Paediatric Respiratory Unit, Great Ormond Street Hospital for Children NHS Foundation Trust, London, UK; 7 Unit of Pediatric Allergy and Respiratory Medicine, Women’s and Children’s Health Department, University Hospital of Padua, Padua, Italy; 8 Department of Maternal, Infantile and Urological Sciences, Sapienza University of Rome, Rome, Italy; 9 Italian Society of Pediatrics (SIP), Rome, Italy; 10 Institute of Translational Pharmacology, National Research Council, Palermo, Italy

**Keywords:** Cigarettes, Smoking cessation, Vaping, ENDS, Adolescents, Quitting, Nicotine dependence

## Abstract

Adolescent nicotine dependence represents a public health challenge, driven by the rapid evolution of tobacco products and by the unique neurobiological vulnerability of the developing brain to nicotine exposure. Clinically significant dependence may emerge after brief and intermittent use, often before adolescents self-identify as users, underscoring the need for proactive and systematic screening across all nicotine and tobacco product modalities. This position paper reviews the evidence on effective cessation strategies for adolescents and provides practical recommendations for clinicians and health systems. Psychological interventions, including cognitive-behavioral therapy and shared decision-making, remain the cornerstone of treatment, given their alignment with the motivational and developmental characteristics of this age group. Educational approaches, particularly school-based and digital interventions, show promise in reducing initiation and supporting cessation among current users. Family engagement and school environment play a critical supporting role, provided that adolescent confidentiality is consistently respected. Pharmacological options are limited by the absence of approved medications for individuals under 18 years of age. However, off-label use of nicotine replacement therapy may be considered in cases of moderate-to-severe dependence under specialist oversight. Effective strategies require integrated care pathways linking primary pediatric care, school health services, and community resources, supported by policy measures that reduce product accessibility and appeal. This joint position paper, endorsed by the Italian Society of Pediatrics (SIP) and the Italian Pediatric Respiratory Society (SIMRI), provides evidence-based recommendations for adolescent smoking cessation, encompassing educational and clinical interventions.

## Introduction

Adolescence is a period of rapid brain maturation, characterized by progressive development of prefrontal control networks and synaptic plasticity [1]. Nicotine exposure during this vulnerable window produces neurochemical and structural changes that potentiate reward sensitivity, impair executive function development, and increase the risk of longer-term addiction and psychiatric vulnerability [2]. Molecular and translational data support nicotine’s capacity to enhance dopaminergic signaling and alter cholinergic receptor expression in adolescent brain regions linked to reward and habit formation [3].

In the United States, 10.1% of high-school students reported past-30-day use of any tobacco/nicotine product, and electronic nicotine delivery systems (ENDS) remained the most common modality among adolescents [4]. Among ENDS, pod-based devices are of particular concern because they can expose youth to higher nicotine concentrations than other tobacco products through the use of nicotine salts, which reduce aerosol harshness and increase palatability at high doses [5]. Tobacco use disorder is a DSM-5–diagnosable substance use disorder; in adolescents, clinically significant dependence may emerge after brief and intermittent use, well before the onset of daily smoking, and often before adolescents self-identify as “smokers” [6]. Adolescent nicotine dependence often presents with patterns distinct from long-term adult smokers: intermittent, social, or device-driven use; preference for flavored products; and concurrent polysubstance use [7]. Clinically relevant dependence may be masked by lower daily consumption but is nevertheless associated with difficulty quitting, withdrawal symptoms, and impaired attention or mood [8]. Screening instruments should be integrated into routine adolescent primary care and school-based health encounters; structured social history must explicitly include ENDS, and specifically differ between heated tobacco products, pod-based vaping and oral nicotine pouches use. The American Academy of Pediatrics recommends that pediatric clinicians routinely screen for nicotine product use and provide anticipatory guidance [9].

This joint position paper, endorsed by the Italian Society of Pediatrics (SIP) and the Italian Pediatric Respiratory Society (SIMRI), provides evidence-based recommendations for adolescent smoking cessation, encompassing educational and clinical interventions.

## Methods

This article is a narrative joint position paper developed by a multidisciplinary panel of Italian and European pediatric experts, endorsed by the Italian Society of Pediatrics (SIP) and the Italian Pediatric Respiratory Society (SIMRI). The manuscript does not follow a systematic review methodology; rather, it is based on a structured narrative synthesis of the literature, supplemented by expert consensus on clinical recommendations. A focused literature search was conducted in PubMed/MEDLINE and Cochrane Library using the following search terms: “smoking cessation adolescents,” “nicotine dependence youth,” “vaping cessation,” “e-cigarette cessation youth,” “tobacco cessation pharmacotherapy adolescents,” “behavioral interventions nicotine adolescents,” “school-based tobacco prevention,” “digital interventions smoking youth,” and “motivational interviewing adolescents.” The search covered publications from January 2000 to April 2026, with priority given to randomized controlled trials, systematic reviews, meta-analyses, and major clinical guidelines. This position paper targets adolescents (< 18 years of age) as its primary population; where evidence is derived from young adult or adult populations, this is explicitly noted. The final document was reviewed and approved by both societies prior to submission.

## The role of pediatricians and healthcare providers

Pediatricians and adolescent health providers are positioned to identify nicotine use early and to offer evidence-based interventions. Core clinical tasks include routine screening, brief motivational counseling, assessment of dependence and comorbidities (mental health, substance use), provision or referral to behavioral cessation programs, consideration of pharmacotherapy when indicated, and systematic follow-up [10]. Brief, developmentally tailored messages delivered by trusted clinicians have demonstrable influence on adolescent attitudes and intentions [9].

Effective cessation requires seamless pathways linking primary pediatric care, school health services, community cessation programs, and specialist adolescent substance-use services [11]. Primary care may adopt simple workflows [12]: (1) ask about all nicotine/tobacco products at every visit involving adolescent (≥ 11 years old); (2) assess readiness to quit; (3) provide brief cessation counseling using motivational interviewing techniques; (4) offer behavioral supports; (5) refer to school- or community-based cessation programs or digital interventions as appropriate; and (6) consider pharmacotherapy in selected cases under specialist oversight. Interdisciplinary case conferences and shared electronic care plans can improve retention and continuity. Clinicians require regular training in cessation methods (motivational interviewing, cognitive-behavioral strategies, pharmacotherapy indications), and systems should provide decision-support tools, referral lists, and quality metrics (screening rates, referral uptake, quit attempts) [13, 14]. Cessation counseling should be conducted confidentially, during one-on-one time without a parent present, to foster candid disclosure and support the therapeutic alliance [15].

Beyond core clinical tasks and care pathways, strengthening pediatric involvement requires targeted training, integrated networks, and active engagement in public health policy. To strengthen pediatricians’ roles, targeted training initiatives should be implemented, focusing on the nuances of adolescent nicotine dependence. This includes understanding the unique patterns of use among adolescents, such as social and intermittent use, and the influence of flavored products on smoking initiation and cessation [9]. Primary care providers should be prepared to address these specific behaviors using tailored counseling techniques. Broader evidence on adolescent smoking cessation highlights the importance of combining behavioral and developmental approaches to improve outcomes [16, 17]. An integrated healthcare network is essential for effective cessation strategies. Collaboration among pediatricians, mental health professionals, and addiction specialists can create a more comprehensive support system for adolescents. Structured referral pathways, shared care processes, and improved coordination across services can support this. Evidence indicates that pediatric providers regularly screen for tobacco use, but they frequently lack systematic connections to cessation services, underscoring the necessity for enhanced integration between clinical care and community resources [18]. A survey conducted in Italy among 1,500 pediatricians identified three profiles (passive, unmotivated, proactive) related to barriers and incentives for smoking cessation counseling among pediatricians, suggesting that tailored educational interventions are required to promote smoking cessation programs [19].

A scoping review of healthcare providers found 50–92% reported some knowledge of vaping but large gaps regarding health effects and cessation methods; only 14% routinely screened adolescents for vaping, and many lacked confidence, time, tools, and clear referral pathways, highlighting the need for guidelines and training [20]. Pediatricians also play a critical role in advocating for policies that restrict access to nicotine products for minors and promote prevention programs in schools. Engaging in local and national advocacy can help shape a healthier environment for adolescents, reducing the overall prevalence of nicotine use [9]. By adopting a proactive and collaborative approach, clinicians may significantly impact adolescent nicotine cessation efforts, leading to improved health outcomes for this vulnerable population.

Italy and the European Union have established specific regulatory frameworks relevant to adolescent nicotine cessation. In Italy, the sale of tobacco and ENDS products is prohibited to individuals under 18 years of age, under Legislative Decree No. 6/2016 (implementing EU Tobacco Products Directive 2014/40/EU) and subsequent national legislation. Despite these restrictions, enforcement at the point of sale remains inconsistent, and youth access to ENDS and flavored nicotine pouches continues to represent a public health concern.

Moreover, Italian public smoking cessation centers are primarily designed for adult smokers, and their accessibility and uptake among adolescents remain limited. In practice, Italian pediatricians are often the first point of contact for adolescent smokers and vapers, particularly during well-child visits or chronic disease follow-up appointments; however, few have received structured training in cessation counseling, as highlighted by a national survey among Italian pediatricians [19].

School health services in Italy may represent an underutilized platform for tobacco/nicotine screening and brief interventions. Future public efforts should expand to cessation-specific resources for adolescents and their families, as well as formal referral pathways from primary pediatric care to addiction and respiratory specialist services.

**Fig. 1:**
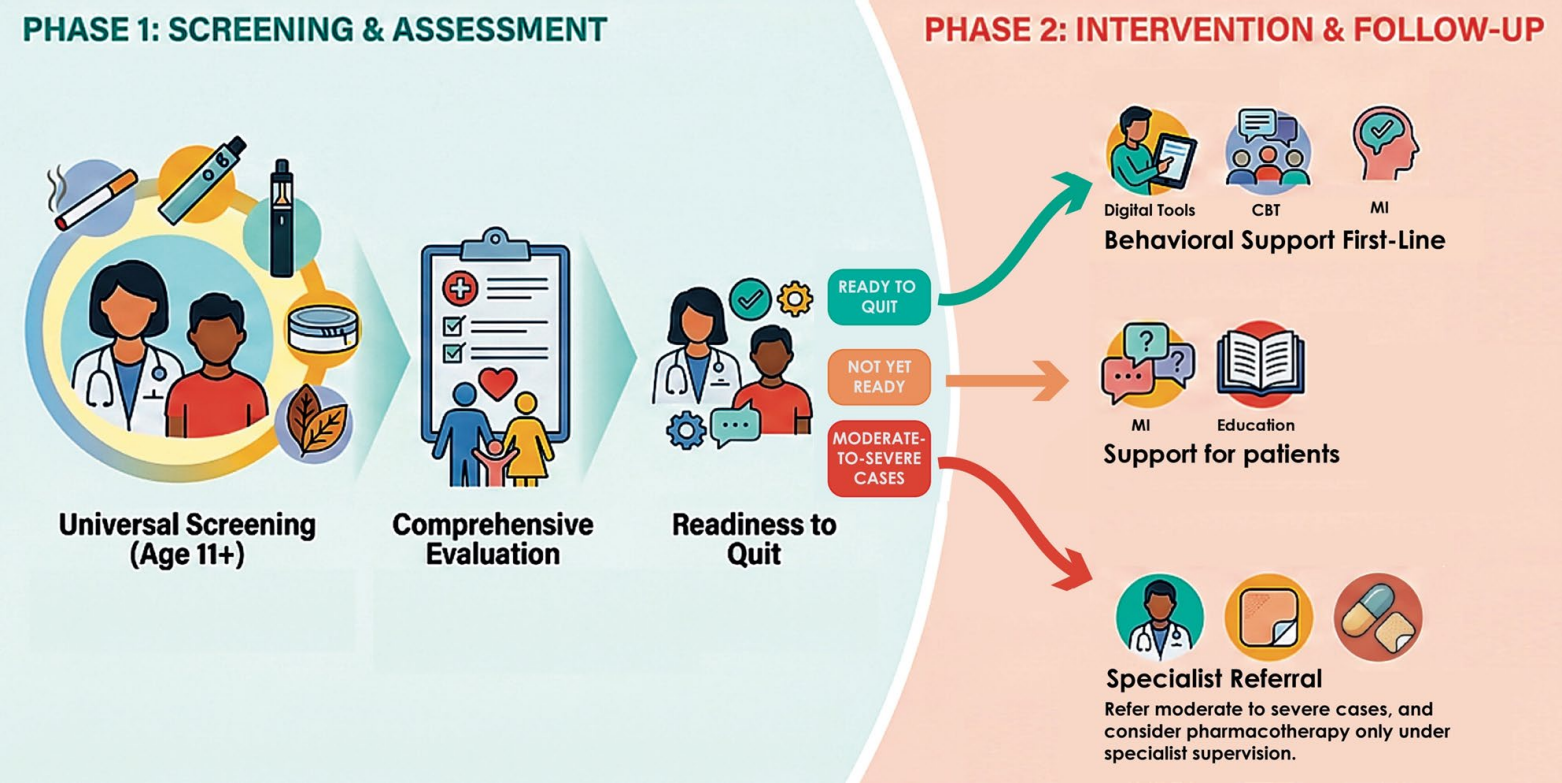
A possible pathway for smoking screening, assessment, and cessation intervention. Abbreviations: CBT = Cognitive-Behavioral Therapy; MI = Motivational Interviewing

Table 1 and Fig. 1 summarize possible recommendations for screening and guiding smoking cessation in adolescents and young adults.

**Table 1:** Recommendations for smoking cessation in adolescents

Intervention	Key Recommendation	Evidence Grade	Applicable Product
*Screening*	Screen all patients ≥ 11 years for all tobacco/nicotine products at every visit	Strong	Cigarettes/tobacco, e-cigarettes/ENDS, oral nicotine pouches
*Counseling*	Deliver brief, non-judgmental cessation advice using Motivational Interviewing; suggest a 2–3 week no-nicotine challenge	Moderate	All products
*Behavioral support*	Combine CBT/MI with digital tools (SMS, web platforms); link all youth to treatment extenders	Moderate	All products
*Pharmacological interventions*	No medication is approved for use under 18 in most jurisdictions; all use is off-label, requires specialist oversight and mandatory behavioral support	Limited	Cigarettes; limited evidence for e-cigarettes/ENDS
*Family engagement*	Counsel parents on tobacco- and aerosol-free homes; offer cessation referral to smoking household members; always respect adolescent confidentiality	Moderate	All products
*Follow-up*	Follow up within 2–3 weeks of quit date; reframe relapse as a learning opportunity	Moderate	All products
*Policy advocacy*	Advocate for flavor bans, age-verification enforcement, advertising restrictions, and tobacco-free schools	Strong	All products

## Psychological interventions

Psychological interventions represent a cornerstone in smoking cessation strategies among adolescents [21]. Adolescents exhibit increased sensitivity to peer influence, emotional regulation challenges, and evolving motivational states, which significantly impact smoking behavior and cessation outcomes. Consequently, interventions targeting psychological mechanisms, such as motivation and social cognition, are particularly relevant in this age group. Among the most widely studied approaches, motivational interviewing has demonstrated consistent, although modest, effectiveness in smoking cessation [22].

Evidence from assessment studies suggests that readiness to quit mediates the effect of interventions on smoking behavior. However, this relationship is strongly influenced by social context, particularly peer smoking. Adolescents with fewer smoking peers show greater responsiveness to interventions targeting motivation, highlighting the interplay between individual psychological readiness and environmental influences and the importance of integrating individual-level psychological strategies with broader social-contextual considerations [23].

Cognitive-behavioral approaches also play a critical role in adolescent smoking cessation by addressing maladaptive thought patterns and enhancing coping skills. These interventions focus on identifying triggers, developing alternative behaviors, and strengthening problem-solving abilities. Emotional regulation is particularly relevant, as smoking in adolescents is often used as a coping mechanism for stress and negative affect. Qualitative evidence indicates that stress, emotional dysregulation, and situational opportunities are major drivers of smoking relapse among vulnerable youth populations, suggesting that interventions targeting coping strategies and distress management are essential [24]. In this context, constructs such as distress tolerance have emerged as important predictors of cessation success.

In addition to traditional face-to-face counseling, digital and web-based psychological interventions have gained increasing attention [25]. These interventions leverage interactive, tailored, and engaging content to enhance user involvement and behavioral change. Qualitative studies of web-based programs for adolescents highlight the importance of personalization, interactivity, and emotional engagement in shaping attitudes and beliefs about smoking. Participants frequently report shifts in risk perception, increased motivation to avoid smoking, and engagement in social discussions about tobacco use, indicating that such interventions may extend their impact beyond the individual to influence social networks [26]. Furthermore, the incorporation of gamification elements and theory-driven frameworks (e.g., transtheoretical model, protection motivation theory) can enhance adherence, self-efficacy, and behavioral learning, thereby improving cessation outcomes [27].

Another promising psychological approach is shared decision-making, which emphasizes patient autonomy and active involvement in treatment choices. In adolescent populations, shared decision-making may be associated with improved motivation, satisfaction, and adherence by aligning interventions with individual preferences and values [28]. By fostering collaborative dialogue between healthcare providers and adolescents, this approach integrates educational, motivational, and behavioral components, creating a more personalized and potentially more effective cessation pathway [29].

Importantly, psychological interventions should not be considered in isolation but rather as part of a comprehensive, multicomponent strategy that accounts for the broader psychosocial environment. Systematic reviews of predictors of smoking cessation among young people consistently identify psychological factors, such as attitudes toward smoking, motivation to quit, and social influences (e.g., peer and family behaviors) as key determinants of cessation success [30]. These findings reinforce the need for interventions that simultaneously target individual cognition, emotional processes, and social dynamics.

## Education, family and technology

It is important to distinguish between evidence on prevention of smoking/vaping initiation among non-users, evidence on cessation support for current users, and evidence on relapse prevention following a quit attempt. These may represent distinct outcomes with partially different evidence bases. Multiple behavioral interventions have been used in adults to support smoking cessation. These include face-to-face counseling, telephone- or video-based call, self-help materials, mobile phone reminders or apps, websites, financial incentives, and exercise-based interventions. Behavioral interventions such as self-printed material and group counselling have been found to be effective. In children and adolescents, although behavioral interventions were associated with a decreased likelihood of cigarette smoking initiation compared with control interventions, no statistically significant difference was observed when these interventions targeted current smokers. Therefore, for smoking children and adolescents, different behavioral interventions should be evaluated [31, 32]. Peer networks remain a dominant predictor of adolescent nicotine uptake and persistence. School climate interventions that reduce peer tolerance for nicotine, amplify prosocial norms, and provide targeted support for marginalized or high-risk students are associated with lower initiation rates. Peer-led campaigns and peer support groups can be powerful adjuncts to clinician-delivered interventions.

Comprehensive school-based interventions that combine curriculum education, policy enforcement (vape-free campuses), and peer-led activities reduce intentions to initiate and can support cessation efforts among current users. A recent Cochrane review and other systematic reviews emphasize that school programs can reduce initiation and support cessation when combined with community measures [33]. Mixed but generally positive effects on knowledge, attitudes, and sometimes behavior have been observed, and programs are more effective when interactive, skills-based, and delivered across multiple years.

Mass-media campaigns and community outreach, when tailored to youth culture and delivered through channels frequented by adolescents (social media platforms, streaming services), can shape norms and reduce product appeal. A trial on social-media delivered anti-vaping campaigns (young adult cohorts) demonstrated promising short-term effects on intention and quitting cognitions [34]. Nonetheless, this trial enrolled young adults rather than adolescents under 18, and while findings are promising, adolescent-specific evaluations of social-media anti-vaping campaigns are not yet available, and generalization to the pediatric age group is premature without dedicated evidence. Education is most effective when combined with policy measures: flavor bans, stricter age verification, retail licensing, taxation, and restrictions on advertising reduce accessibility and “de-normalize” nicotine use among youth. Schools should be supported by local policy to enforce tobacco- and vape-free environments, including prevention of covert use (disposable vapes, small devices) [4].

Parental smoking and vaping are among the strongest predictors of adolescent nicotine initiation and persistence. Household tobacco- and vape-free rules, parental monitoring, and modeling nicotine-free behaviors are associated with reduced youth experimentation. Clinicians should frame nicotine dependence to parents as a medical condition with effective treatments, reducing parental defensiveness and supporting a collaborative family-centered approach. Households should be advised to remove all tobacco products and devices from the home, and caregivers who smoke or vape should be offered referral to adult cessation services, framing a simultaneous household quit attempt as a powerful modeling opportunity. Parental engagement should always be offered but never imposed: adolescents who do not wish to involve their parents in their cessation treatment must have their decision respected in accordance with the principles of confidential adolescent care, and tobacco-use status should not be disclosed in after-visit summaries when confidentiality has been requested. Interventions that include family sessions demonstrate increased cessation attempts and reduced re-initiation in some trials [35]. While parents could play an important role in smoking prevention, there is no conclusive evidence of an effect when they are involved in smoking cessation studies [36]. From a clinical standpoint, parental engagement should always be offered but never imposed. Adolescents who do not wish to involve their parents in their cessation treatment must have their decision respected, in accordance with the principles of confidential adolescent care; clinicians should ensure that tobacco-use status is not disclosed in the after-visit summary or patient portal when confidentiality has been requested. A practical approach is to provide adolescents with written educational materials about nicotine dependence and cessation resources that they can voluntarily share with their families.

Best practice models integrate family engagement and school support through standardized referral routes from schools to primary care cessation services, family education modules, and school-based cessation groups. These integrated models reduce barriers (transportation, stigma) and increase engagement among adolescents.

An Instagram-based randomized controlled trial is evaluating a 5-week vaping cessation program for Californian adolescents and young adults aged 13–21 years, with biochemically verified abstinence and reductions in vaping as primary outcomes. If proven effective, this intervention could represent the first evidence-based Instagram-delivered intervention for vaping cessation in this population [37]. A randomized controlled trial found that adding a text message component to an internet-based smoking cessation program did not improve 9-month abstinence rates among adult smokers in the United States compared with an internet-only program, even though participants reported higher engagement and satisfaction with the combined intervention at 3 months [38]. By contrast, a meta‑analysis of 13 randomized trials (27,240 participants ≤ 30 years) found that stand‑alone SMS programs significantly improved continuous abstinence versus inactive controls (RR 1.51, 95% CI 1.24–1.84), with effects persisting, though attenuated, to 6 months; app‑based interventions showed no clear efficacy, based on sparse and biased data [39]. It should be noted that the majority of trials included in this meta-analysis enrolled participants up to 30 years of age, and findings may not be fully generalizable to adolescents under 18. Adolescent-specific evidence on mobile phone-based cessation interventions remains scarce, and direct extrapolation from young-adult trials should be made with caution.

A study conducted on Swedish high school students found no significant effects of text messaging on abstinence at 6 months, largely due to high dropout rates, although analyses suggested effects in the expected direction [40]. Studies of smoking cessation interventions targeting high school students should include strategies to improve adherence during medium- and long-term follow-up. A systematic review of 14 cessation studies (18–26 years) reported effective but heterogeneous interventions, including SMS, social media, web/apps, counseling, pharmacotherapy, and self‑help; no single modality was clearly superior, underscoring the need for direct comparative trials [41]. App‑based protocols are now testing just‑in‑time cognitive‑behavioral and mindfulness/acceptance messages to reduce urges and smoking in high‑risk situations [42]. Digital tools have also been applied to tobacco–cannabis co‑use, using ecological momentary assessment and multicomponent web/app interventions to characterize co‑use patterns and support dual cessation [43]. Overall, SMS-based interventions show promise for young people, consistently with the unpredictable progress of technology which could make them an obsolete tool, but further research with standardized protocols and longer follow-up is needed.

A systematic review of nicotine replacement therapy (NRT) in adolescents reported limited long‑term cessation efficacy but consistent reductions in smoking frequency and withdrawal symptoms, with generally mild adverse effects, supporting NRT as an adjunct to intensive behavioral support rather than a stand‑alone solution for this age group [44]. A systematic review and meta‑analysis of six randomized trials (10,192 participants) showed that behavior‑based, non‑pharmacologic school programs significantly reduced smoking initiation at six months (RR 0.38), with diminishing effects at longer follow‑up; culturally tailored, peer‑led and combined interventions were most effective [45]. Co‑designed animated videos increased perceived harms of e‑cigarettes and of mixing alcohol with antidepressants, while being highly rated for quality, relevance, and usefulness, illustrating the impact of youth‑led digital resource development [46]. A systematic review of predictors of adolescent cessation identified 63 factors across sociodemographic, psychosocial, behavioral, social, environmental, smoking‑related, health, and genetic domains, implying that effective interventions must address both individual and contextual determinants [47]. Anti‑smoking media campaign exposure was associated with higher 30‑ and 90‑day cessation among adult smokers, with similar benefits across sex, race/ethnicity, income, and education; however, the absence of differential effects suggests that broad campaigns alone may not reduce disparities without added targeting [48].

## Pharmacological interventions

Nicotine addiction commonly begins during adolescence, a developmental period in which brain is particularly vulnerable to nicotine exposure. However, adolescents recognize when smoking or vaping is becoming an addiction and many try to stop on their own [49]. Notably, smoking cessation for subjects in this age range may be particularly difficult, since adolescents face not only the effects of nicotine deprivation, but also concerns about parents’ and peers’ opinions, especially those who smoke. In addition, they may lack the resources to obtain cessation aids or may be reluctant to seek help. As a consequence, there is a higher risk of relapse in adolescents than in adults [44, 50].

Pharmacological strategies for smoking cessation are commonly used in adults motivated to quit. Evidence shows that combining pharmacotherapy with behavioral strategies is more effective than either approach alone [51]. These products aim to reduce withdrawal symptoms by targeting nicotine dependence and can be divided into “long-acting” and “short-acting”. Daily long-term maintenance doses stabilize the neurochemical effects of nicotine dependence and should be taken regularly to prevent withdrawal symptoms and reduce craving throughout the day. These therapies include long-acting forms of NRT, such as transdermal patches, as well as non-nicotine oral medications like bupropion and varenicline. Bupropion is an atypical antidepressant and was the first non–nicotine-based pharmacotherapy approved for smoking cessation. Bupropion acts by weakly inhibiting the reuptake of norepinephrine and dopamine and partially blocking nicotine receptors, thus reducing nicotine cravings. Varenicline, instead, works as a partial agonist of the alpha4-beta2 nicotine receptor, thereby reducing cravings, withdrawal symptoms and the rewarding effects of smoking [51]. In some European countries, cytisine/cytisinicline, a plant-derived alkaloid, is used as a long-acting therapy, acting similarly to varenicline as a partial agonist of nicotine receptors [52].

In contrast, short-acting therapies are treatments that should be used to manage acute cravings and the urge to smoke; examples are nicotine gums, lozenges, inhalers, and nasal sprays, which deliver nicotine more rapidly. Combining a long-acting with a short-acting seems to be a better strategy than using a single therapy alone, especially when integrated with behavioral support. Varenicline is currently considered the most effective pharmacotherapy for smoking cessation and therefore the American Thoracic Society recommends varenicline over bupropion and NRT [53].

Few studies have evaluated the efficacy and safety of these products in young people and adolescents. For this reason, unless otherwise specified, pharmacological evidence cited in this section derives from trials that variously enrolled adolescents (12–17 years), young adults (18–25 years), or mixed cohorts. A Cochrane review included 41 trials involving more than 13,000 people under 20 years of age and found that both behavioral support and smoking cessation medication increased the proportion of young people quitting smoking in the long-term, but evidence was insufficient to support a specific pharmacological strategy [54]. A systematic review including 1,188 adolescent smokers aged 12–20 years found that pharmacotherapy increases abstinence rate but only in the short-term, probably due to adherence and behavioral factors [55]. Nevertheless, a more recent review involving 5,122 subjects aged 11–21 years and smoking at least 1 cigarette per day showed that the use of NRT for a treatment duration ranging from 6 to 12 weeks, administered as inhaler, chewing gum or patch, with nicotine patches being the most common form of therapy, did not achieve significant rates of tobacco abstinence at the end of treatment in the majority of studies, but the products were well tolerated [44]. Notably, most of the studies show that use of NRT and other drugs for smoking cessation in young subjects seems to have poorer outcomes than in adult trials, but many protocols didn’t follow the same dose or length of treatment used in adults and study protocols are heterogeneous. However, the adverse events reported in adolescents in these studies are similar to those reported in adults for NRT, bupropion and varenicline [56].

No medication is currently approved for subjects under 18 years of age in most high-income jurisdictions, including the United States, the European Union, and the United Kingdom; the regulatory situation may differ in other countries. All pharmacological use in this age group is therefore off-label and must be contextualized accordingly. The American Academy of Pediatrics supports considering off-label NRT in selected adolescents with moderate-to-severe nicotine dependence and motivation to quit [9, 57]. Before initiating NRT or any other pharmacotherapy in an adolescent, clinicians should: (1) formally assess nicotine dependence using a validated instrument; (2) confirm genuine motivation to quit and willingness to engage in concurrent behavioral support; (3) review contraindications (active cardiovascular disease, pregnancy, hypersensitivity); (4) address parental or legal guardian notification where legally or ethically required, while respecting adolescent confidentiality to the extent permitted; (5) ensure specialist adolescent medicine or addiction medicine oversight; and (6) establish mandatory concurrent behavioral support, as pharmacotherapy alone has not demonstrated sustained efficacy in this population.

In the World Health Organization guidelines, it is emphasized that there is insufficient evidence to recommend routinely prescribing or recommending cessation medications for adolescents, since their effectiveness has not been consistently demonstrated although they are generally safe [58].

A randomized clinical trial compared 12 weeks of double-blind varenicline vs. placebo, each added to remotely delivered behavioral counseling and compared with single-blind enhanced usual care, with monthly follow-up to 24 weeks. The 261 participants (16–25 years old), vaped nicotine daily or near daily, but did not regularly smoke tobacco and wanted to reduce or quit vaping. The results show that varenicline increased the proportion of participants achieving 4-week continuous vaping abstinence at the end of treatment (51% vs. 14%) [59].

Further corroborating evidence has recently emerged from a meta-analysis, which specifically addressed the gap in FDA labeling for varenicline use in youth [60]. The authors conducted a random-effects meta-analysis of five RCTs enrolling a total of 717 nicotine users aged 12 to 26 years, including both combustible cigarette smokers and ENDS users. Varenicline demonstrated a statistically significant increase in abstinence rates compared to alternative interventions including placebo, bupropion, and nicotine patch (RR 1.99; 95% CI 1.04–3.80), and a similarly significant advantage over placebo. Importantly, the safety profile of varenicline was comparable to that of placebo with respect to treatment-emergent adverse events, and to other pharmacological comparators with respect to treatment discontinuation events. Subgroup analyses restricted to adolescents alone or to combustible cigarette users only yielded smaller, non-significant effect estimates, highlighting that the overall efficacy signal was driven substantially by young adults and ENDS users, and that age and type of nicotine exposure may modulate treatment response. Nonetheless, given the small number of included trials, moderate-to-high heterogeneity in some analyses, and limited long-term follow-up data, these results should be interpreted with caution and do not yet alter the recommendation that varenicline use in subjects under 18 remains off-label and requires specialist oversight combined with mandatory behavioral support.

## Conclusion

Adolescent nicotine dependence demands a proactive, adolescent-tailored response from pediatricians, school health services, families, and policymakers. Behavioral and psychological interventions remain the cornerstone of treatment; pharmacotherapy has a selective and closely supervised role. Digital tools and social-media campaigns offer scalable engagement opportunities but require adolescent-specific evaluation. Structural policy measures, such as flavor bans, age-verification enforcement and tobacco-free school environments, are essential complements to clinical care. Robust longitudinal research and integrated surveillance systems will support the continuous refinement of interventions in parallel with the rapidly evolving landscape of nicotine products.

## Data Availability

Not applicable.
